# Supplementary material for: MetaRibo-Seq measures translation in microbiomes
Source: Nat Commun. 2020 Jun 29;11:3268. doi: 10.1038/s41467-020-17081-z (PMC7324362; doi:10.1038/s41467-020-17081-z)
Supplement: Supplementary file 10 — Supplementary Data 7 [file 41467_2020_17081_MOESM10_ESM.zip › File2/Confidence_High_Taxonomy/371425_out.krona.html]

Javascript must be enabled to view this page.

members
magnitude
magnitudeUnassigned
count
unassigned
taxon
rank

371425\_out

3

superkingdom
3
2

1239
phylum
3

186801
class
3

186802
order
3

1392389
genus
1


SRS144537\_contig\_number\_contig-100\_2020.268516
1
species
1297617

186803
2
family

1506553
2
genus

species
1

SRS019910\_contig\_number\_contig-100\_990.126477
1512

1

SRS143895\_contig\_number\_16685
species
1871021
